# Enhancing reginal wall abnormality detection accuracy: Integrating machine learning, optical flow algorithms, and temporal convolutional networks in multi-view echocardiography

**DOI:** 10.1371/journal.pone.0310107

**Published:** 2024-09-12

**Authors:** Sazzli Kasim, Junjie Tang, Sorayya Malek, Khairul Shafiq Ibrahim, Raja Ezman Raja Shariff, Jesvinna Kaur Chima

**Affiliations:** 1 Faculty of Medicine, Universiti Teknologi MARA (UiTM), Sungai Buloh Campus, Sungai Buloh, Malaysia; 2 Cardiac Vascular and Lung Research Institute, Universiti Teknologi MARA (UiTM), Shah Alam, Malaysia; 3 National Heart Association of Malaysia, Heart House, Kuala Lumpur, Malaysia; 4 Faculty of Medicine, Cardiology Department, Universiti Teknologi MARA (UiTM), Shah Alam, Malaysia; 5 Faculty of Science, Bioinformatics Division, Institute of Biological Sciences, University of Malaya, Kuala Lumpur, Malaysia; Al-Nahrain University, IRAQ

## Abstract

**Background:**

Regional Wall Motion Abnormality (RWMA) serves as an early indicator of myocardial infarction (MI), the global leader in mortality. Accurate and early detection of RWMA is vital for the successful treatment of MI. Current automated echocardiography analyses typically concentrate on peak values from left ventricular (LV) displacement curves, based on LV contour annotations or key frames during the heart’s systolic or diastolic phases within a single echocardiographic cycle. This approach may overlook the rich motion field features available in multi-cycle cardiac data, which could enhance RWMA detection.

**Methods:**

In this research, we put forward an innovative approach to detect RWMA by harnessing motion information across multiple echocardiographic cycles and multi-views. Our methodology synergizes U-Net-based segmentation with optical flow algorithms for detailed cardiac structure delineation, and Temporal Convolutional Networks (ConvNet) to extract nuanced motion features. We utilize a variety of machine learning and deep learning classifiers on both A2C and A4C views echocardiograms to enhance detection accuracy. A three-phase algorithm—originating from the HMC-QU dataset—incorporates U-Net for segmentation, followed by optical flow for cardiac wall motion field features. Temporal ConvNet, inspired by the Temporal Segment Network (TSN), is then applied to interpret these motion field features, independent of traditional cardiac parameter curves or specific key phase frame inputs.

**Results:**

Employing five-fold cross-validation, our SVM classifier demonstrated high performance, with a sensitivity of 93.13%, specificity of 83.61%, precision of 88.52%, and an F1 score of 90.39%. When compared with other studies using the HMC-QU datasets, these Fig s stand out, underlining our method’s effectiveness. The classifier also attained an overall accuracy of 89.25% and Area Under the Curve (AUC) of 95%, reinforcing its potential for reliable RWMA detection in echocardiographic analysis.

**Conclusions:**

This research not only demonstrates a novel technique but also contributes a more comprehensive and precise tool for early myocardial infarction diagnosis.

## Introduction

Coronary artery disease, which can lead to severe myocardial ischemia and infarction (MI), is a leading cause of mortality worldwide [1]. Diagnosing MI involves integrating clinical data with biochemical markers like serum levels, electrocardiogram (ECG) results, and various imaging modalities to assess cardiac muscle function [2]. Both early detection and prompt intervention are essential to prevent further damage to heart tissue and reduce the risk of death [3]. Accurate diagnosis of MI relies on the estimation of myocardial motion, particularly of the left ventricle (LV), which is currently performed qualitatively and remains dependent on the interpreter’s judgment [4]. Among the earliest and most reliable indicators of MI is the regional wall motion abnormality (RWMA) in the ventricular muscle. Regional wall motion abnormality is not only an early signal of cardiovascular diseases such as MI but also a critical marker of systolic ventricular function, which reflects cardiac wall motion and regional contractile function [5].

Transthoracic echocardiography (TTE) is frequently employed as a first-line diagnostic tool in individuals suspected of MI, owing to its cost-effectiveness and non-invasive nature compared to cardiac magnetic resonance imaging (MRI) and computed tomography (CT) scans[6]. TTE is an ultrasound-based diagnostic test that produces live images of the heart, known as echocardiograms [4]. Multiple views are commonly utilized, including the parasternal windows in both short-axis and long-axis orientations, as well as the apical windows, which are typically represented by the apical two-chamber (A2C) and apical four-chamber (A4C) views echocardiograms [7]. Myocardial function is typically quantified by the left ventricular ejection fraction (LVEF), which is usually calculated from both the A2C and A4C views echocardiograms [7]. Assessing RWMA is also essential for a comprehensive visual evaluation of global myocardial function from both the A2C and A4C views as well[3]. Although echocardiography is a prevalent diagnostic modality, its efficacy in evaluating myocardial function heavily relies on the experience and skill set of the operator, as well as the interpretive expertise of the cardiology specialist. Hence, interpretation of RWMA remains to be operator and interpreter dependent and is often gauged relative to either normal neighboring myocardial segments (if available) or based on the individual operator and interpreter experience if there is global dysfunction throughout the organ [8,9]. For example, when a cardiologist visually evaluates the motion activity of an A4C view echocardiogram, the infarcted segments that show a ‘‘reduced” motion (or almost no motion at all) compared to other segments [10].

Integrating machine learning (ML) and deep learning (DL) algorithms into echocardiography enhances the accuracy of myocardial function assessments, enabling automated analysis and reducing operator dependency. These advancements facilitate precise segmentation and quantification of cardiac parameters, improving diagnostic capabilities. LV wall structure segmentation, based on cardiac wall motion estimation algorithms or DL-based semantic segmentation algorithms, provides clear LV contour for further quantified measurement. Cardiac wall motion estimation algorithms, pivotal, utilize pixel-based or block-based tracking to analyze LV wall displacement in echocardiogram frames, thereby offering crucial quantitative cardiac parameters, such as global longitudinal strain (GLS), LVEF, myocardial mass, chamber sizes, and identification of systolic and diastolic phases [3,7,11,12]. Traditional computer vision and signal processing algorithms employed for this purpose include Active Polynomials (APs) [10], optical flow [11], sparse representation [13], and the Difference of Gaussian (DOG) pyramid algorithm [14]. DL-based semantic segmentation algorithms, such as U-Net [7,9], and Graph Convolution Neural Network (GCN) [15], have significantly advanced the extraction of quantitative cardiac parameters, including myocardial mass, LVEF, and regional wall displacements. These parameters are crucial for various CVDs diagnoses when used in conjunction with ML classifiers [3,5,16,17]. U-Net, in particular, has enhanced biomedical image analysis, outperforming conventional ML algorithms [18] and spurring a series of studies on DL’s role in echocardiography segmentation [5,7], that provides precise LV wall segments.

The detection of CVDs incorporates a range of computational strategies, leveraging the latest advancements in ML and DL. These methodologies are broadly categorized into three strategic approaches: (1) the deployment of end-to-end DL classifiers on echocardiogram images, (2) multi-stage analysis of image features, and (3) multi-stage analysis that integrates quantified cardiac measurements with ML classifiers [6]. End-to-end Coevolution Neural Network (CNN) architectures, such as 3D CNNs for MI detection using time-series echocardiography videos, have achieved notable accuracy levels, averaging at 84.6% [8,19]. Self-attention fusion network (SAF-Net) is applied to detect MI with a self-attention mechanism to learn dependencies in extracted feature vectors from the A4C view and A2C view [17]. Time-series analysis with Long Short-Term Memory (LSTM) Networks has shown the image features extracted by convolution layers from key frames in systolic or diastolic phases in MI detection from echocardiogram frames compared to traditional Artificial Neural Networks (ANNs) [20]. Multi-stage analysis mostly relies on accurate instance segmentation of LV wall to provide quantified cardiac parameter measurement as input features to classifiers, such as LV segment intersections, to assess regional myocardial function [12]. In GLS analysis, the LV contours segments displacement curves are generated based on annotation of 2D echocardiography videos. Then, the peak values of displacement curves, obtained during systolic and diastolic phases, are utilized in as motion information for supervised ML classifiers, such as Support Vector Machines (SVM) and Random Forest (RF), and K-Nearest Neighbors (KNN) for the binary detection of RWMA or other CVDs [3,5,7,16].

Despite the advancements in employing DL and ML for automated CVDs diagnosis in echocardiograms, significant challenges persist. A primary challenge is the scarcity of publicly available datasets with CVD labels, such as the EchoNet-Dynamic [21], the CAMUS [22], and the CETUS dataset from the MICCAI challenge 2014. The HMC-QU dataset is a comprehensive dataset released for RWMA identification in the heart’s LV, yet its exclusivity also limits the diversity of data used in studies [5]. Additionally, there are issues related to the performance of the motion estimation algorithms, such as gradient-based optical flow methods in ultrasound imaging, which varies significantly with the angle and depth of the ultrasound beam, resulting in image artifacts that hinder accurate motion estimation [23–25]. These can be improved by DL segmentation using trained U-Net [16,17]. Another concern is the reliance on the feature extracted from specific phases (end diastolic, mid systolic, and end systolic) in one echo cycle echocardiograms only [20], such as the motion feature in most DL studies with GLS analysis are defined as the peak value of endocardial boundary points displacement curves. Thus, although some studies have attempted to fused the motion features from both A4C and A2C views, the performance of single view and muti-views are similar [16,17]. Consequently, this limitation suggests that the quality of these parameters might not be satisfied for accurate ML or DL classifier performance. Furthermore, while previous research has underscored the usefulness of time-series features and optical flow algorithms in tracking cardiac motion from echocardiogram tissue velocity images [11,23–25], a DL algorithm that can learn from cardiac wall motion fields features has yet to be reported. This gap indicates an area for future exploration and development in the field. Despite some studies attempting to fuse motion features from both apical A4C and A2C views, the performance of single-view and multi-view analyses remains similar [3,16]. This similarity in performance indicates that the quality of these parameters may not be adequate for accurate ML or DL classifier performance. Moreover, while previous research has emphasized the benefits of time-series features and optical flow algorithms for tracking cardiac motion from echocardiogram tissue velocity images [11,23–25] a DL algorithm capable of learning from cardiac wall motion field features has not yet been developed. This gap highlights a significant area for future research and development in the field.

Hence, our study introduces a novel approach for the diagnosis of RWMA by combining ML classifiers with DL feature extraction, using optical flow motion fields as features inputs. These motion fields, generated by optical flow algorithms, enable our method for capturing subtle movements of the cardiac wall among serval echo cycle, which are crucial for detecting RWMA. We employ optical flow algorithms to map cardiac wall movement to cardiac wall motion fields. This technique allows us to capture dynamic cardiac activities across consecutive echocardiogram frames, offering insights into myocardial function that were previously unattainable with conventional static algorithms. In our analysis, we leverage U-Net to provide precise boundaries of cardiac structures and remove the noise in the background. The accurate segmentation provided by U-Net significantly improves the quality of subsequent motion field mapping, ensuring that the motion vectors used in our analysis are both accurate and meaningful. Furthermore, we have adapted a Temporal Segment Algorithm (TSN), traditionally used in video recognition [26], to process these optical flow motion fields within echocardiogram analysis. This adaptation allows for the utilization of motion vectors as robust features for detecting RWMA, representing a novel strategy in cardiac imaging. Extending beyond standard applications, our methodology incorporates a Temporal Convolutional Network (ConvNet), inspired by the Temporal Segment Network [26]. This ConvNet is crucial for identifying object movement patterns from the optical flow-based data [27], a method not previously reported in the CVDs diagnosis. Our approach is structured as a three-phase algorithm that begins with U-Net segmentation coupled with optical flow analysis to examine cardiac wall motion field features comprehensively. Following this, the Temporal ConvNet divides a video into segments, processes each segment independently, and aggregates these features to form a complete video-level representation. This representation effectively classifies various action categories, indicating different stages or instances of RWMA. For the implementation of our Temporal ConvNets, we selected the ResNet101 architecture [28] due to its proven efficacy in enhancing feature extraction through deep residual learning. This choice underscores our commitment to utilizing robust architectures that complement our innovative approach. The final stage of our methodology involves the classification of the extracted motion field features using a suite of DL and ML classifiers, including SVM, RF, KNN, Decision Trees (DT), and Neural Networks (Multi-layer perceptron, MLP). This integrated approach marks a significant departure from existing studies, which primarily focus on echocardiogram segmentation using conventional optical flow algorithms [29–32]. By innovatively applying these techniques to RWMA detection through detailed cardiac motion analysis, our study introduces significant advancements in the accuracy and capability of diagnostic tools for cardiac health, establishing a new frontier in medical imaging technology.

## Materials and methods

### Overview of proposed method

**Fig 1:**
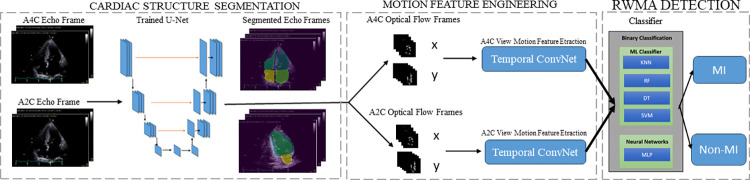
The steps of proposed method of RWMA detection using echocardiography images for RWMA detection, consisting with cardiac structure segmentation using a trained U-Net on both A4C and A2C echo frames, resulting in segmented echo frames; motion features engineering with optical flow frames in both the A4C and A2C views, with backbone Temporal ConvNets to analysis the X and Y components of the motion; RWMA detection, using extracted features are fed into a suite of ML classifiers—including KNN, DT, Random Forest, SVM, and MLP—to achieve binary classification for the presence of RWMA or non-RWMA conditions.

The proposed three phases algorithm framework for the binary RWMA detection is explains in Fig 1. This process simplifies the complex staging of myocardial segments on the LV wall by categorizing them into a binary system. This method aligns with other peer-reviewed studies [5,10,16]. The approach includes the segmentation of cardiac structures, extraction of motion features, and the actual detection of RWMA.

**Cardiac Structure Segmentation:** a three-beat two-dimensional loop of 2 standard view echocardiograms is extracted and converted into multiple frames, each with a resolution of 224×224 pixels. This loop was then divided into multiple frames. The trained U-Net [7] was then used as a segmentation Network on the ordered echocardiogram images to segment the different cardiac chambers. The echocardiograms were composed of the segmented echocardiograms images after segmentation stage using the trained U-Net.

**Motion Feature Engineering:** After segmentation, the dense optical flow method analyzes the frames to extract movement features from the consecutive echocardiogram frames. This method determines the direction and velocity of the moving cardiac wall, which is vital for understanding myocardial motion using the OpenCV-Python package [OpenCV. (2015). Open-Source Computer Vision Library].

**RWMA Detection:** The processed frames with optical flow information from both A2C and A4C views are then inputted into Temporal ConvNets. These Networks, which are pre-initialized with ImageNet weights and fine-tuned with A2C and A4C view data from the HMC-QU dataset, specialize in analyzing time-related features. They generate a feature vector of 2048×1 dimensions.

Finally, these features are classified using multiple ML classifiers, including SVM, KNN, DT, RF, and MLP, with five-fold cross-validation (CV) method to determine whether RWMA is present. This multistage approach effectively combines structural and motion data to assess the heart’s wall motion, aiming to accurately identify abnormalities.

### The HMC-QU dataset

**Fig 2:**
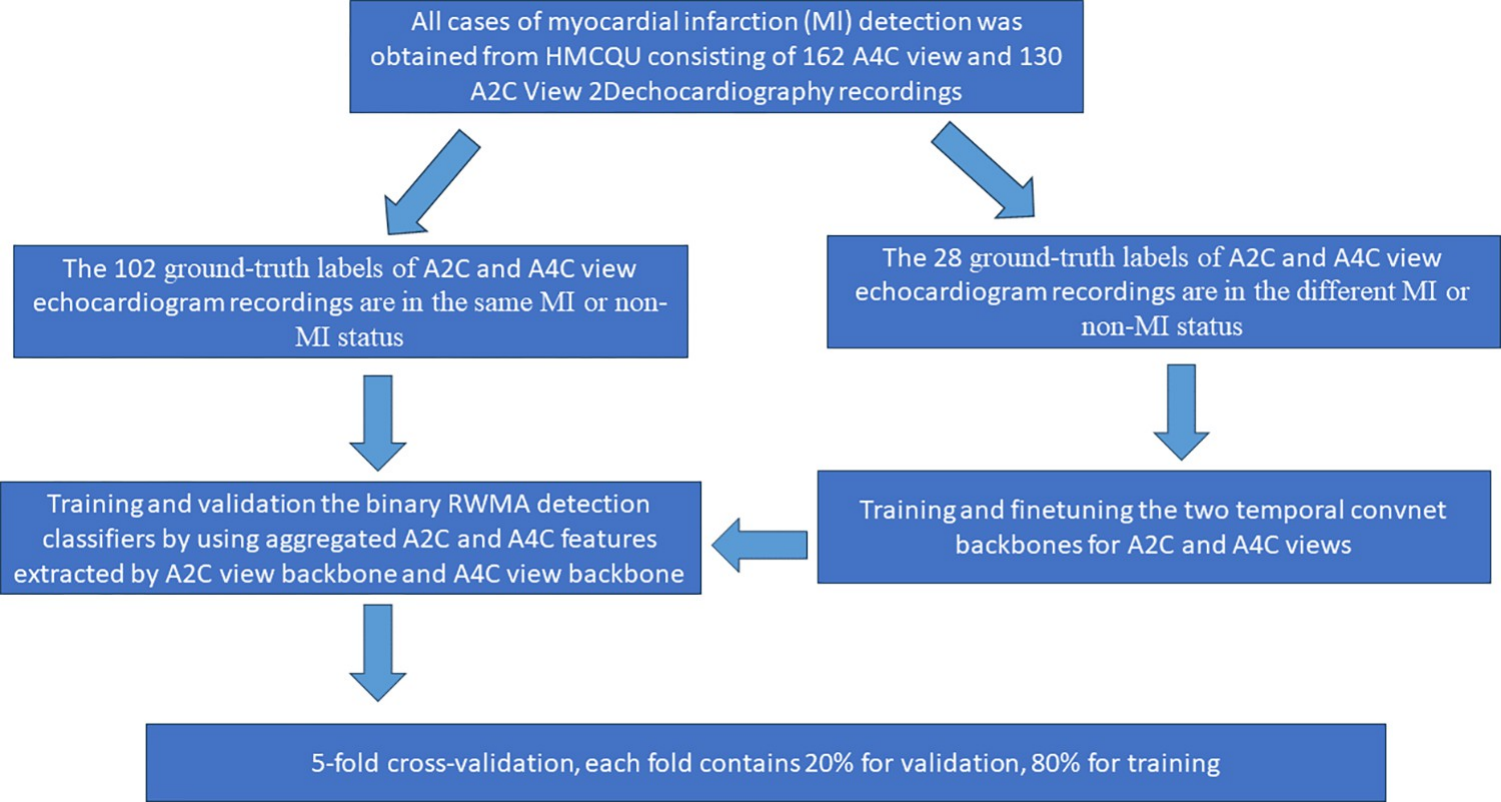
Selection of the study population from the HMC-QU dataset for temporal ConvNet backbones finetune and RWMA detection classifiers training and validation.

The HMC-QU dataset, developed by Hamad Medical Corporation (HMC), Tampere University, and Qatar University, is the first public dataset made available to the scientific community. This dataset has received ethical approval from the local ethics board of HMC Hospital as of February 2019. It caters to both MI diagnosis and LV wall segmentation [10].The dataset includes a collection of A4C and A2C view of 2D echocardiography recordings obtained during the years 2018 and 2019. Each echocardiogram in the HMC-QU dataset consists of around 25 frames. In this study, we perform a binary classification task to simplify the problem. Therefore, we have downsized the ground-truth labels to 1−non-RWMA (normal), and (2, 3, 4, 5) − RWMA. In Fig 2, HMC-QU dataset consists of 162 A4C view 2D echocardiography recordings, including 93 MI subjects (all first-time and acute MI) and 69 non-MI subjects. Also, the dataset consists of 130 A2C view 2D echocardiography recordings that belong to 79 MI subjects and 51 non-MI subjects. It means MI ratios are 61.54% and 52.3% in A4C and A2C views that the ratios differ from each other since only 60 subjects have their MI visible in both views. Therefore, in multi-view echocardiography, we considered the ground-truth labels correspond to 88 MI subjects and 42 non-MI subjects, where the ground-truth labels are formed as MI if any of the views depict RWMA, whereas non-MI if no sign of RWMA is visible in both views. Thus, we divided the HMC-QU dataset into two parts according to whether the ground-truth labels echocardiogram recordings are in the same status in Table 1.

**Table 1:** The number of subjects with respect to their corresponding ground-truth labels from A4C and A2C views.

Ground-truths A4C view A2C view			Subjects number
A4C view	A2C view	Subject label	
MI	MI	RWMA	60
non-MI	non-MI	non-RWMA	42
MI	non-MI	RWMA	20
non-MI	MI	RWMA	8

Fig 2, Illustrates the study data splitting methods for the HMC-QU dataset, which comprises 162 A4C view and 130 A2C view 2D echocardiogram recordings. These recordings are essential for the detection of MI. Ground-truth labels are assigned to 102 of these recordings, ensuring that both A2C and A4C views consistently display the same MI or non-MI status, which forms the primary dataset for the initial training phase. For the fine-tuning phase, an additional subset of 28 echocardiogram recordings is employed. These recordings consist of A2C and A4C views with differing MI statuses, presenting a diverse dataset. This diversity is essential for enhancing the temporal Convents’ capacity to detect small variances in cardiac motion, which may indicate the presence or absence of MI. The next stages involve training and validating binary RWMA detection classifiers. These classifiers utilize aggregated features from A2C and A4C view echocardiographic data extracted by the temporal ConvNet backbone. The classification process employs a five-fold CV method to optimize the models’ performance, ensuring robustness and generalizability. Each fold of the comprises 20% of the data reserved for validation and 80% for training purposes, aligning with standard practices to prevent overfitting. Specifically, the subset of 28 echocardiograms displaying divergent MI statuses across views is crucial for fine-tuning the temporal ConvNet backbones tailored to A2C and A4C views. This subset ensures that the ConvNets are sensitive to variances in cardiac motion patterns associated with different stages and severities of MI. Additionally, the larger subset of 102 echocardiograms with MI and non-MI statuses across views supports the training and validation of the binary RWMA detection classifiers. This division of the dataset into training and fine-tuning phases underpins the efficacy of the RWMA detection process.

### Cardiac structure segmentation by U-Net

**Fig 3:**
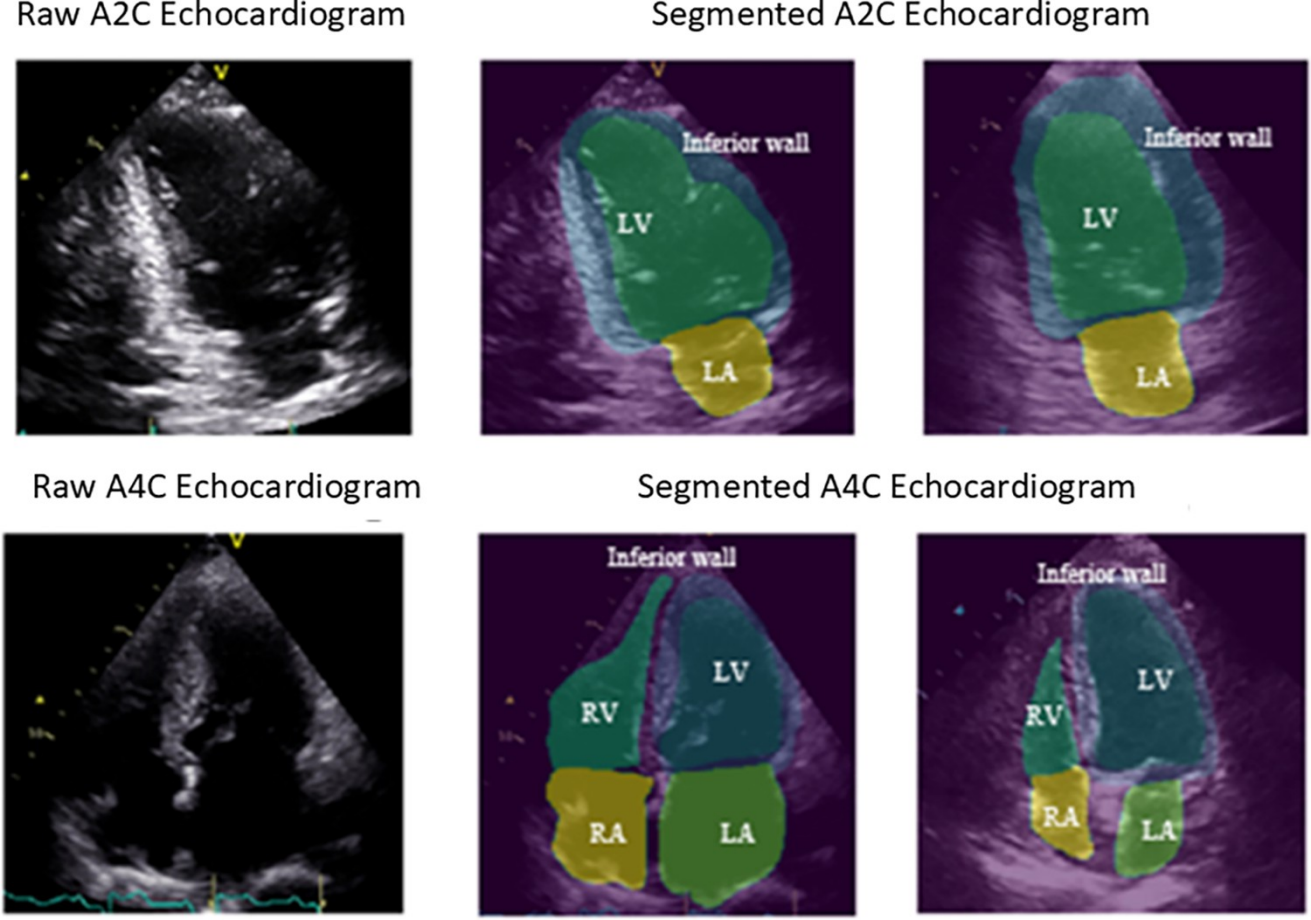
ROI of cardiac structure in echocardiograms generated by U-Net.

Accurate extraction of the LV wall is crucial to obtain the true motion of the myocardium. We first used U-Net [18] to segment the frames extracted from the HMC-QU echocardiograms for because there is no available public dataset for the whole heart structures chambers with masks label, and only the LV ground truth masks were provided by the HMC-QU dataset. The U-Net used in this study was trained based on the dataset constructed from a total of 14, 035 echocardiograms obtained from the University of California San Francisco (UCSF) server. This dataset was specifically curated for the purpose of detecting hypertrophic cardiomyopathy and cardiac amyloidosis [7]. U-Net is a DL algorithm composed of a contracting path (left side) and an expanding path (right side) with a total of 23 convolutional layers. The contracting path follows the typical architecture of a convolutional network. It consists of the repeated application of two 3x3 convolutions (unpadded convolutions), each followed by a rectified linear unit (ReLU) and a 2x2 max pooling operation with stride 2 for down sampling. Consequently, the expansive path is more or less symmetric to the contracting path and yields a U-shaped architecture [18]. Separate U-Net Networks were used to perform segmentation task on different views frames including A4C, and A2C views raw echocardiograms to generate corresponding ROI (regions of interest). Fig 3 depicts the result obtained from this stage utilizing unprocessed echocardiography data. The echocardiography (Fig 3) reveals the anatomical composition of the heart chambers, including the LV, Left Atrium (LA), Right Ventricle (RV), Right Atrium (RA), and Inferior Wall. After the segmentation performed using U-Net the A2C and A4C echocardiograms as seen in Fig 3 highlighted cardiac chambers ROI on single frame was created. Fig 3 displays the instance segmentation results generated by the pre-trained U-Net used in our study. We also evaluated the pre-trained U-Net performance by the pixel-by-pixel comparison of LV ground truth masks and the predict masks of the corresponding echocardiogram from the pre-trained U-Net.

**Fig 4:**
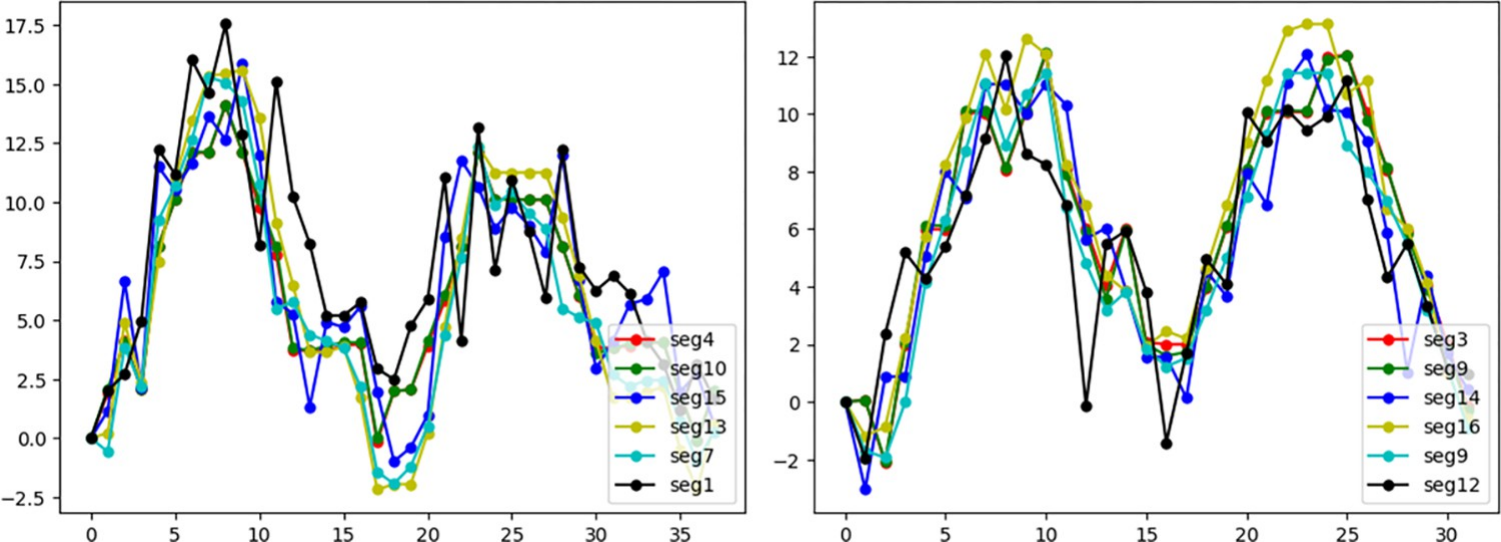
(a) A2C and (b) A4C displacements curves recordings of a patient consist of two cardiac cycles from A2C and A4C echocardiograms.

In Fig 4, the U-Net is employed to extract the LV contour in each frame of the echocardiography recordings and the LV contour length is segmented into left and right parts, as previously explained, with the left part denoted as *L* and the right part as *R*. Following this extraction, the LV boundary is tracked, and its displacement compared with the first frame of the echocardiogram is measured across several cardiac cycles. In the A2C and A4C views, the length of the apical myocardial segments is represented as *R*/7 for the right part and *L*/7 for the left part. In contrast, the other myocardial segments are represented as 2*R*/7 and 2*L*/7 for the right and left parts, respectively. It illustrates the displacement curves for each myocardial segment in both A2C and A4C view echocardiograms, representing two cardiac cycles. These displacements are calculated over the cardiac cycles recorded. Each curve corresponds to a specific segment of the heart, capturing the motion from end-diastole to end-systole. The curves depict how different heart segments move relative to each other, with their maximum displacements highlighted. These plots are instrumental in understanding how different sections of the heart move relative to each other, providing vital data that inform the classification models used in the study. The selection of maximum displacements helps in identifying potential areas with abnormal motion, which could indicate the presence of RWMA.

### Optical flow data generation

**Fig 5:**
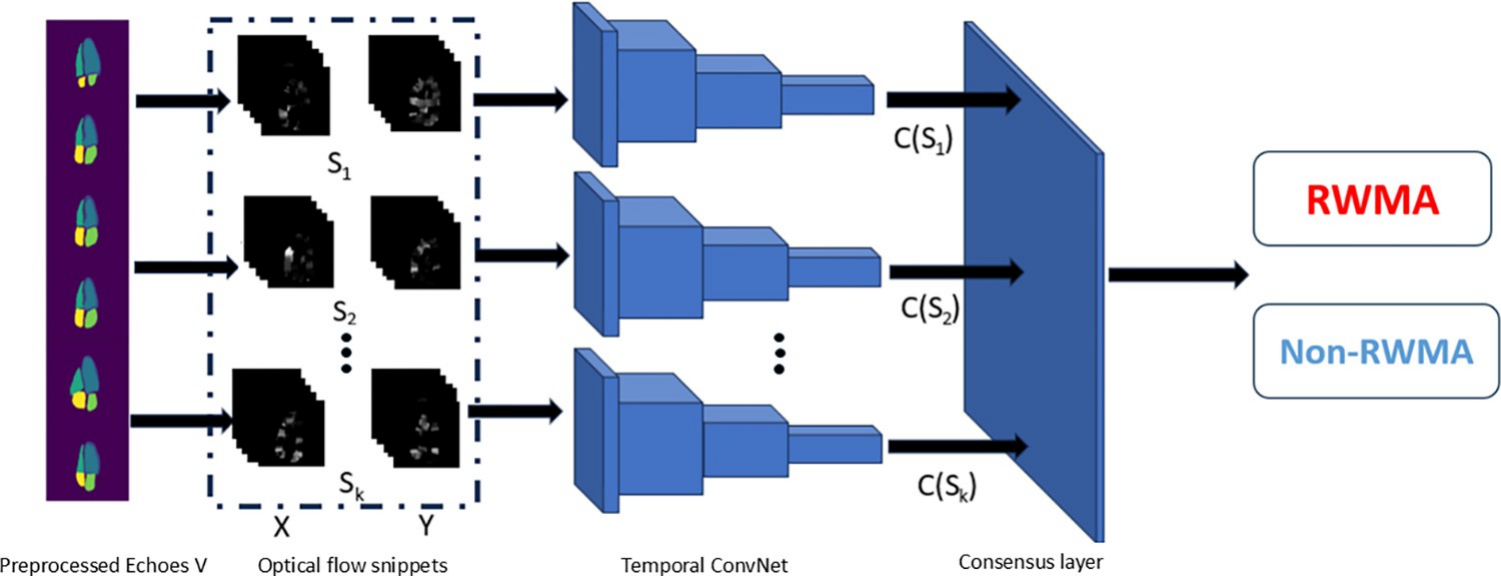
The motion feature extraction part for the Temporal ConvNet backbone employed for echocardiograms. Each of the *K* optical flow format echocardiogram snippets is classified using one Temporal ConvNet instance. The horizontal(X) and vertical(Y) Optical flow fields were extracted 5 frame each time. The first convolution layer is adjusted to 224×224×10 to accept. The snippet-level echocardiograms c (s;) are aggregated in the consensus layer, yielding the overall RWMA detection. Snippets are stacks of several optical flow fields calculated between consecutive echocardiogram frames. Temporal ConvNets backbone on all snippets share same parameters.

The RWMA can be visualized by echocardiograms and quantified measured by the cardiac parameter GLS with the maximum values of LV counter segments displacement curves in previous study [10]. In our study, we utilize dense optical flow algorithm to estimate cardiac wall movement. Optical flow fields effectively highlight the differences in motion between healthy subjects and those with RWMA, providing a visual representation of regional heart function disparities. The optical flow snippets in Fig 5 were generated in this study from the consecutive frames from segmented echocardiograms that illustrates the clear boundary of heart chambers ROI. The motion field of image objects between two consecutive frames caused by the movement of an object in the window is known as optical flow. The motion field was extracted from segmented echocardiograms with distinct chamber structures using the optical flow method with A2C view and A4C view. Fig 5 depicts the optical flow of segmented echocardiograms in both horizontal and vertical views.

We choose the dense optical flow algorithm [27] implemented in OpenCV. The dense optical flow estimation assumes that the brightness of a moving pixel remains constant over time between consecutive frames. And neighboring pixels have similar motion. Briefly, optical flow estimation by two frames is formulated as follows:

$$E_{t}(u,v)=F(I_{t},I_{t+1};\theta F)$$ (1)

where *I*_*t*_ and *I*_*t+1*_ are two successive segmented echocardiogram frames, which separately denote the current frame at time *t* (i.e. the target frame) and the next frame at time *t*+1 (i.e. the neighboring frame). *F* is the function utilized to calculate the dense optical flow motion field in the previous video recognition study using optical flow motion field and *ΘF* represents its parameters [28]. *E*_*t*_(*u*, *v*) denotes the optical flow motion field, as well as displacement vector in frame *t*, with the horizontal and vertical flow components *u* and *v* respectively. The resulting optical frames in vertical and horizontal directions were saved based on RWMA label and view categories. Then, the temporal ConvNet framework extracts short snippets over a long optical flow echocardiogram sequence with a sparse sampling scheme, where the samples distribute uniformly along the temporal dimension.

### Motion feature engineering

**Fig 6:**
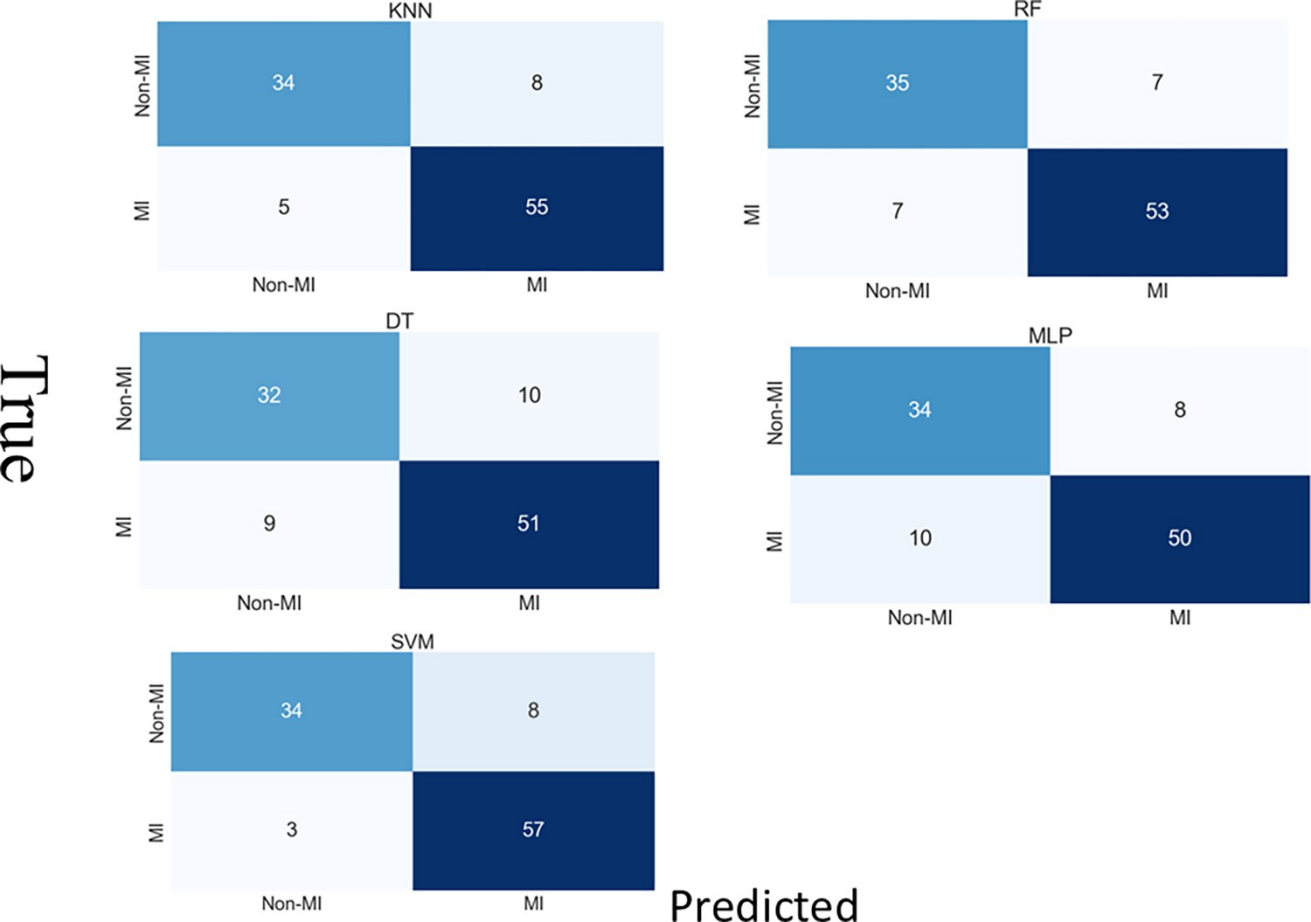
Overall confusion matrices of all classifiers used in our study for RWMA detection performance results (multi-vies) with the optimized parameters by grid search computed over the test sets of 5−fold in HMC-QU dataset.

In this study we utilize temporal ConvNet as backbone to learn the optical flow motion field features from the entire echocardiogram video to perform RWMA prediction. Fig 6 showed the motion feature extraction part based on the Temporal ConvNet. The training of temporal ConvNet is under the 28 Subjects’ ground-truth labels of A2C and A4C view echocardiogram recordings are in the different RWMA or non- RWMA status. We adapt the original ResNet 101 architecture [28] as the base of Temporal ConvNets. First, this framework in Fig 5 extracts short snippets over a long optical flow echocardiogram sequence with a sparse sampling. Then, a segmental structure is employed to aggregate information from the sampled snippets. Temporal ConvNet takes a stack of *E*_*t*_(*u*, *v*) as input. The first convolution layer of Temporal ConvNet is adjusted to 224×224×10 to accept the optical flow data. In this part, the length of the optical flow fields as input feature vectors with the shape 224×224×10 as one snippet containing 5 frames of optical flow snippets from the same echocardiogram, that 5 vertical frames and 5 horizontal frames. These vectors are then input into the Temporal ConvNet, where they undergo convolution and pooling operations that transform the vector’s dimensions. The final length of the feature vector that reaches the consensus layer for classification is 1024×1, specifically the number and size of the convolutional filters and the extent of pooling applied across temporal segments. Instead of working on single frames or frame stacks, temporal segment Networks operate on a sequence of short snippets sparsely sampled from the entire echocardiogram. Each snippet in this sequence will produce its own preliminary prediction of the RWMA detection. Formally, given a echocardiograms V, we divide it into *k* segments {*S*_1_, *S*_2_, …, *S*_*k*_} of equal duration. Then, the temporal segment Network models a sequence of snippets as follows:

$$TSN(T_{1},T2,\ldots ,Tk)=H(g(F(T_{1};W),F(T_{2};W),\ldots ,F(T_{k};W))))$$ (2)

Here (*T*1, *T*2, …, *TK*) is a sequence of snippets. Each snippet *T*_*k*_ is randomly sampled from its corresponding segment *S*_*k*_. *F*(*T*_*k*_; *W*) is the function representing a Temporal ConvNets with parameter *W* which operates on the short snippet *T*_*k*_ and produce class scores for all the classes. The segmental consensus function g combines the outputs from multiple short snippets to obtain a consensus of class hypothesis among them. The segmental consensus function *G* combines the outputs from multiple short snippets to obtain a consensus of class hypothesis among them, in our experiment snippets number *k* = 7 showed the best performance.

The loss function used is SoftMax function for H. Combining with standard categorical cross-entropy loss, the final loss function *L* regarding the segmental consensus $G=g(F(T_{1};W),F(T_{2};W),\ldots ,F(T_{k};W))$ is formed as where C is the number of action classes and *yi* the ground truth label concerning class *i*.


$$L(y,G)=-\overset{C}{\underset{i=1}{\sum }}y_{i}(G_{i}-log\overset{C}{\underset{j=1}{\sum }}exp\;G_{j})$$ (3)

### RWMA detection

The cardiac motion features generated from the Temporal ConvNet in this study were then utilized for the diagnosis of regional RWMA. In order to achieve this, we have employed traditional ML and MLP algorithms to identify RWMA based on the extracted cardiac motion features from echocardiograms. The RWMA binary classification task utilized supervised algorithms, including DT, RF, SVM, KNN, and MLP. The output of the last max pooling layer of the Temporal ConvNet is a 1024×1 matrix representing the cardiac motion features. These features are retrieved from the A4C and A2C views videos separately. The feature vector for each view is concatenated together to generate a 2048×1 shape vector. Next, these features were fed into the classifiers to detect the RWMA.

DT uses a hierarchical tree structure for effective classification. RF, an enhancement of DT, addresses overfitting by utilizing an ensemble of trees. SVM robustly classifies data via a hyperplane, leveraging its kernel trick for optimized separability. KNN classifies data samples based on the majority class of its ’k’ nearest points. DL, or MLP, captures intricate data relationships through iterative training, fine-tuning for tasks like discerning RWMA in echocardiograms [33].

To further refine our models, we adopted a GRID search methodology. This technique entails a comprehensive exploration over a pre-defined hyperparameter space. By evaluating model performance across this vast grid, our endeavor was to unearth the ideal hyperparameters that amplify predictive prowess. Such rigorous calibration ensures our models’ resilience and fine-tuning to our dataset’s unique attributes. The classifiers are evaluated in a stratified 5-fold CV scheme for fair performance evaluation. Their configuration, training and testing details are explained in the next section.

## Performance evaluation

The performance evaluation of the proposed approach is carried out for RWMA detection only in independent 102 subjects’ samples of the HMC-QU dataset as in Fig 3 of ROI of cardiac structure in echocardiograms generated by U-Net. The main objective of the analysis is to obtain the highest possible sensitivity, with a reasonably high specificity, in order to not miss any subject with RWMA. For the RMWA detection, we consider the infarcted class, RWMA, as class-positive and normal, non-RWMA as class-negative. In this case, the confusion matrix is formed as; TN is the number of correctly predicted non-RWMA subjects, TP is the number of correctly predicted RWMA subjects, FN is the number of incorrectly detected RWMA subjects as non-RWMA subjects, and FP is the number of incorrectly detected non-RWMA subjects as RWMA subjects. Also, the Area Under the Curve (AUC) of the Receiver Operating Characteristic (ROC) curve is a vital metric for disease classification. This curve, plotted with False Positive Rate (FPR) and True Positive Rate (TPR) at various thresholds, effectively quantifies diagnostic accuracy. The confusion matrix elements are calculated per-video for the MI detection. The standard performance evaluation metrics are defined as follows.


$$TPR=\frac{TP}{P}=\frac{TP}{TP+FN}$$ (4)

True Positive Rate (TPR or sensitivity) is the ratio of correctly detected positive samples to all positive class members,

$$FPR=\frac{FP}{FP+TN}$$ (5)

False Positive Rate (FPR) is the ratio of incorrectly detected positive samples to all negative class members

$$SPE=\frac{TN}{TN+FP}$$ (6)

SPE (specificity) is the ratio of correctly detected negative samples to all negative class members,

$$P=\frac{TP}{TP+FP}$$ (7)

P (precision) is the rate of correctly predicted positive class members in the all members detected as a positive class,

$$F1=\frac{2TP}{2TP+FP+FN}$$ (8)

F1 is the harmonic average of precision and sensitivity,

$$ACC=\frac{TP+TN}{TP+TN+FP+FN}$$ (9)

Accuracy (ACC) is the rate of all the correctly predicted classes among all the data. Accuracy might be a misleading performance metric when the dataset is imbalanced.

Also, the CV method was used to evaluate the overall performance of the constructed model as published studies in RWMA detection using the HMC-QU dataset [3,5,16,19]. All datasets were initially divided into several groups, one of which was used as the test group for the evaluation, while the remaining data were used for training. Thereafter, the accuracy was comprehensively calculated by repeating the process such that all data are test data. In this study, we used a five-fold CV method for comprehensive compassion in which the echocardiography videos with random sampling so that all cases were used as test data.

## Results

In this study, we used a pretrained U-Net for Cardiac structure segmentation. Figs 3 and 4 displays the segmentation outcomes from the pre-trained U-Net model on the HMC-QU dataset, with high accuracy yet lower recall rates across 5 different folds as detailed in Table 2, and the displacement curves in two echo cycles. Table 2 depicts the results of pre-trained U-Net and gained the overall accuracy in 97.16% in total 2349 frames, suggesting the model is adept at identifying negative cases over positive ones in a binary classification context.

**Table 2:** The Performance of pre-trained U-Net model on the HMC-QU dataset.

CV FOLD	SENSITIVITY	PRECISION	F1-SCORE	ACC
**fold1**	58.90%	69.10%	62.92%	97.02%
**fold2**	58.31%	72.46%	64.08%	97.19%
**fold3**	55.17%	72.43%	61.85%	97.38%
**fold4**	57.29%	69.62%	62.27%	96.97%
**fold5**	55.35%	72.31%	61.85%	97.29%

**Fig 7:**
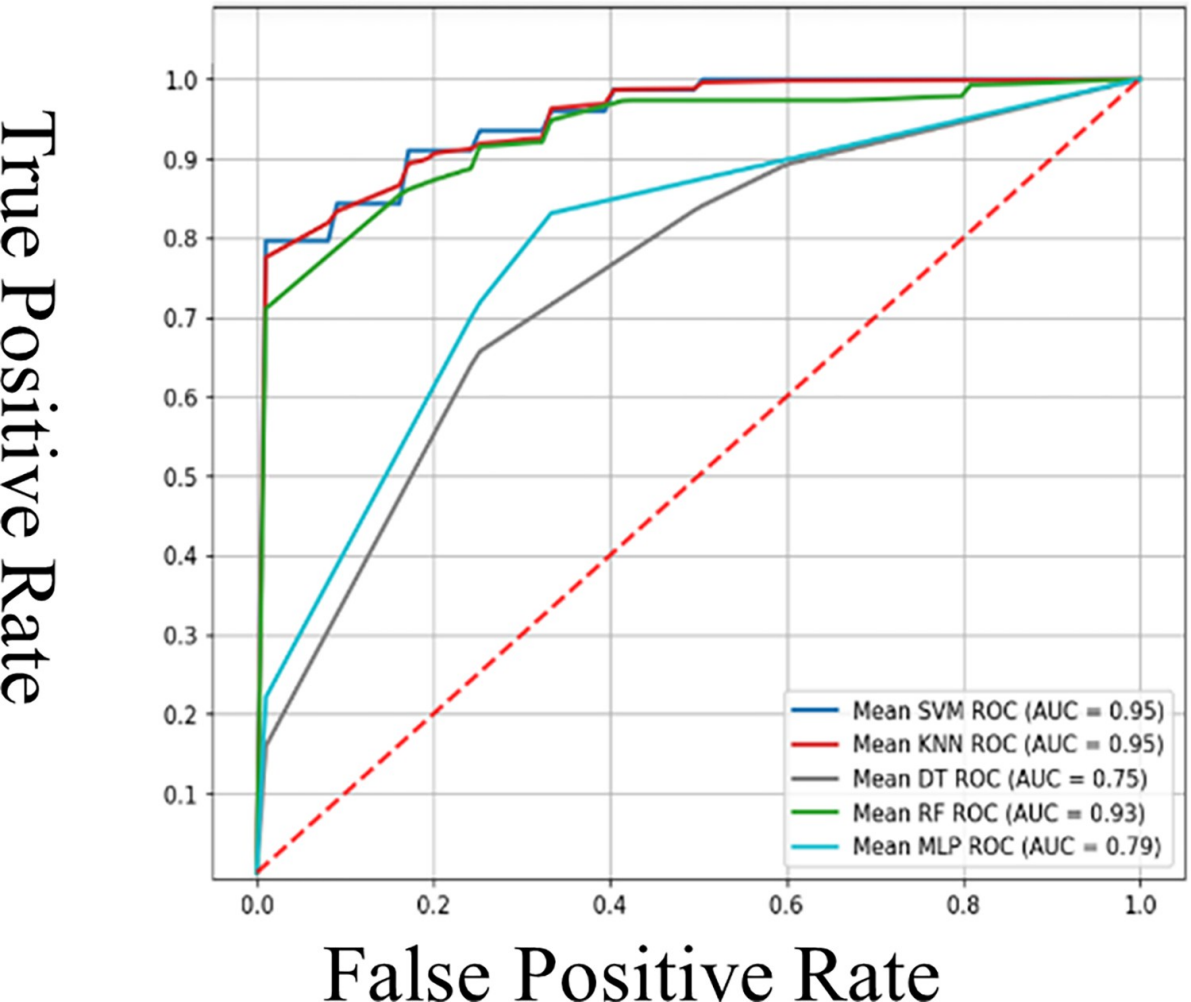
Mean AUC for RWMA detection performance results (multi-vies) computed over the test sets of 5−fold in HMC-QU dataset.

ML methods and MLP algorithms for the RWMA detection, utilizing motion features derived from A2C, A4C, and multi-view echocardiograms. As detailed in the methodology section, these features were processed using classifiers, including DT, RF, SVM, and MLP. This analysis used a five-fold CV, drawing upon the best parameter configurations identified through grid search. In Table 3, our primary metric was the confusion matrices and AUROC for early RWMA detection, showcased in Figs 6 and 7. Secondary performance indicators included average values for accuracy, sensitivity, specificity, and the F1 score, all of which are tabulated in Tables 4–6. In evaluating the performance of various ML models for the detection of RWMA across different views in the HMC-QU dataset, our analysis reveals that KNN and SVM models generally exhibit high sensitivity and precision, with SVM achieving 100% maximum sensitivity in several instances but with variable minimum values. KNN shows consistent performance with strong F1 scores, suggesting a reliable balance between precision and sensitivity. MLP stands out for its specificity and precision in certain conditions, yet exhibits significant variability across different test sets, indicating potential stability issues. RF and DT present moderate performance, with RF outperforming DT in general but not reaching the consistency of KNN or SVM. Notably, the A4C view provides better model performance than the A2C view, which might indicate a more reliable view for RWMA detection or better model tuning for this view. This analysis underscores the importance of model selection based on clinical priorities and the necessity of comprehensive CV to ensure the dependability of RWMA detection in clinical applications. The classifier shows high accuracy but low recall across all folds, indicating it is better at predicting the negative class than the positive in a binary classification task. The Fig 6 illustrates the overall confusion matrices for multiple classifiers used in the study for the detection of RWMA using the HMC-QU dataset across a five-fold validation setup. Each matrix represents a different classifier, specifically KNN, RF, DT, SVM, and MLP. The cells in each matrix indicate the number of predictions for each class.

**Table 3:** The average RWMA detection performance results (multi-views) with the optimized parameters by grid search.

CLASSIFERS	SENSITIVITY	SPECIFICITY	PRECISION	F1 SCORE	ACC	AUC
*KNN*	91.67%	80.95%	87.30%	89.43%	87.25%	95.00%
*DT*	85.00%	76.19%	83.61%	84.30%	81.37%	75.00%
*RF*	88.33%	83.33%	88.33%	88.33%	86.27%	93.00%
*SVM*	95.00%	79.07%	86.36%	90.48%	89.22%	95.00%
*MLP*	83.33%	80.95%	86.21%	84.75%	82.35%	79.00%

**Table 4:** Overall RWMA detection performance results (multi-views) computed over the test sets of each 5−fold in HMC-QU dataset.

	*Sensitivity*			*Specificity*			*Precision*			*F1 score*			*ACC*		
	*Max*	*Mean*	*Min*	*Max*	*Mean*	*Min*	*Max*	*Mean*	*Min*	*Max*	*Mean*	*Min*	*Max*	*Mean*	*Min*
**SVM**	100.00%	90.86%	87.50%	100.00%	79.67%	75.00%	100.00%	88.20%	40.00%	96.00%	89.13%	77.70%	95.24%	89.25%	80.00%
**RF**	100.00%	83.03%	76.92%	100.00%	74.03%	66.67%	100.00%	83.03%	63.63%	94.12%	81.71%	73.68%	85.71%	82.38%	75.00%
**KNN**	100.00%	87.14%	84.61%	93.30%	77.33%	66.67%	100.00%	87.14%	87.50%	94.73%	86.19%	77.78%	95.24%	89.24%	80.00%
**DT**	100.00%	92.60%	69.23%	100.00%	64.47%	66.67%	100.00%	82.13%	75.00%	87.50%	85.73%	81.81%	80.95%	78.62%	76.19%
**MLP**	100.00%	80.60%	46.15%	100.00%	81.40%	66.67%	100.00%	79.83%	70.00%	94.74%	80.24%	63.15%	95.24%	87.19%	65.00%

**Table 5:** Average RWMA detection performance results (A4C view) computed over the test sets of each 5−fold in HMC-QU.

	*Sensitivity*			*Specificity*			*Precision*			*F1 score*			*ACC*		
	Max	Mean	Min	Max	Mean	Min	Max	Mean	Min	Max	Mean	Min	Max	Mean	Min
SVM	100.00%	82.16%	19.51%	100.00%	78.61%	54.54%	100.00%	83.67%	46.34%	94.59%	79.21%	32.60%	90.24%	72.77%	19.51%
RF	95.23%	83.24%	60.97%	100.00%	74.82%	54.54%	100.00%	85.21%	62.96%	93.15%	82.18%	72.72%	87.80%	75.29%	60.98%
KNN	95.24%	83.24%	51.22%	100.00%	74.83%	50.00%	100.00%	84.34%	59.26%	94.59%	81.68%	67.74%	90.24%	76.09%	51.22%
DT	95.24%	78.68%	53.66%	100.00%	64.20%	50.00%	100.00%	84.43%	64.00%	90.41%	79.67%	69.84%	82.93%	71.74%	53.66%
MLP	95.23%	83.09%	58.54%	100.00%	77.26%	50.00%	100.00%	86.79%	62.07%	91.67%	83.02%	72.72%	87.80%	76.36%	58.53%

**Table 6:** Average RWMA detection performance results (A2C view) computed over the test sets of each 5−fold in HMC-QU.

	*Sensitivity*			*Specificity*			*Precision*			*F1 score*			*ACC*		
	Max	Mean	Min	Max	Mean	Min	Max	Mean	Min	Max	Mean	Min	Max	Mean	Min
**SVM**	100.00%	77.44%	58.97%	100.00%	62.94%	40.00%	100.00%	80.34%	46.34%	89.19%	75.88%	63.33%	80.49%	63.29%	40.00%
**RF**	90.48%	76.56%	66.67%	100.00%	60.27%	47.50%	100.00%	82.13%	57.69%	87.67%	77.67%	66.67%	78.05%	67.29%	47.50%
**KNN**	89.47%	82.02%	69.23%	100.00%	64.66%	50.00%	100.00%	82.59%	64.29%	89.19%	80.79%	73.47%	80.49%	74.32%	68.29%
**DT**	95.24%	78.68%	53.66%	100.00%	64.20%	50.00%	100.00%	84.43%	64.00%	90.41%	79.66%	69.84%	82.93%	71.74%	53.66%
**MLP**	94.74%	69.62%	15.38%	100.00%	67.63%	18.18%	100.00%	81.78%	47.06%	87.67%	70.83%	26.09%	87.50%	65.63%	17.07%

In this comparative analysis of classifiers for detecting MI from echocardiographic images, the KNN and SVM classifiers exhibit standout performances. KNN demonstrated high sensitivity, identifying a substantial number of True Positives (55) while minimizing False Negatives (5), crucial for medical diagnostics were failing to detect MI can result in critical outcomes. On the other hand, SVM not only matched the high sensitivity of KNN by correctly identifying 57 MI cases, but it also maintained a low rate of False Negatives (3), making it an exceptionally reliable tool for MI detection. The DT and MLP classifiers, while useful, displayed more mixed results. DT had a higher rate of False Positives (10) and False Negatives (9), suggesting it may be less effective in environments where precision is paramount. Similarly, MLP, with False Positives (8) and False Negatives (10), indicates a potential for improvement in both sensitivity and specificity. In contrast, the RF classifier showed excellent specificity, with the highest number of True Negatives (35) and the lowest False Positives (7), indicating its strength in accurately ruling out non-MI cases. However, its slightly higher False Negatives (7) compared to KNN and SVM might be a concern in critical diagnostic settings.

## Discussion

Table 2 shows the multi-views results of evaluation metrics, by each classifier using 5-fold CV calculated with their corresponding test sets. Our experimental findings have demonstrated that the SVM and KNN (AUC = 95%) classifier outperforms other algorithms DT, RF, and MLP in terms of AUC as shown in Fig 7 and Table 2. However, SVM performs better than KNN when other performance metrics are taken into account 89.25% accuracy, 90.48% F1 score, 86.36% precision, 79.07% specificity, and 95.00% sensitivity. The results obtained from our study are consistent with those obtained from other studies that employed SVM as a classifier when utilizing the HMC-QU dataset [3,5,16]. This reaffirms the robustness of SVM in handling datasets like HMC-QU and its potential as a leading algorithm in RWMA detection [16,33].

Table 7 demonstrates the comparison with similar study using the HMC-QU dataset. A review of related works using the HMC-QU dataset has shown varied performance in terms of different computational strategies used in RMWA disease detection task. In summary, RMWA detection using single view reported lower accuracy using the HMC-QU dataset and k-fold CV in studies conducted by Serkan Kiranyaz, 2022 (ACC = 83.13%) [10], Mohamed Saeed, 2022 (ACC = 80.22%) [19], Aysen Degerli‬, 2021 (ACC = 80.24%) [5], and 2023 (ACC = 85.38%) [17] compared to results obtained from our study using the SVM classifier (ACC = 89.25%). Hamila et al.,(2022) used 3D CNN methods, including R2D and R3D CNNs, that have been tailored for binary RWMA detection [8]. Also, SAF-Net used fused features from the A4C and A2C view with an accuracy of 78.13% in 10-fold CV [17]. Cardiac parameters such as GLS are used automatic RWMA detection. For instance, RWMA disease classification using the GLS calculated from the single A4C view reported lower accuracy in studies conducted by Serkan Kiranyaz (2020), with an accuracy of 83.13% without train-test split or k-fold validation, and Aysen Degerli (2021) [5], who achieved an accuracy of 80.24% with 5-fold CV by replacing the segmentation algorithm with U-Net [18]. The DL based segmentation algorithm, LVSnake achieved an accuracy of 83.09% with 5-fold CV.

**Table 7:** Comparison of related works by using HMC-QU dataset.

CLASSIFIER	SENSITIVITY	SPECIFICITY	PRECISION	F1 SCORE	ACC	VIEW	AUTHOR
**ACTIVE POLYNOMIALS (APS)**	89.01%	75.36%	82.65%	85.71%	83.13%	A4C	Serkan Kiranyaz, 2020[10]
**ECHO CARDIO 3D-NET (EC3D-NET)**	81.82%	81.82%	81.82%	81.82%	78.26%	A4C	G Sanjeevi,2023 [34]
**3DCNN**	85.00%		89.00%	87.00%	85.00%	A4C	Oumaima Hamila,2022 [8]
**LVSNAKE(GCN-BASED)**	90.11%	0.00%	86.49%	87.17%	83.09%	Fused A2C & A4C	Yuxuan Li, 2023 [16]
**LDA**	81.38%	72.32%	86.61%	83.21%	78.52%	A2C	Aysen Degerli‬,2021‬[5]‬‬‬‬‬‬‬‬‬‬‬‬‬‬‬‬‬‬‬‬‬‬‬‬‬‬‬‬‬‬‬‬‬‬‬‬‬‬‬‬‬‬‬‬‬‬‬‬‬‬‬‬‬‬‬‬‬‬‬‬‬‬‬‬‬‬‬‬‬‬‬‬‬‬‬‬‬‬
**DT**	79.09%	62.60%	81.72%	80.00%	73.53%		
**RF**	82.29%	67.37%	84.94%	82.65%	76.61%		
**SVM**	83.09%	74.03%	74.03%	84.83%	80.24%		
**SAF-NET**	77.64%	79%	88.26%	81.57%	78.13%	Fused A2C & A4C	Ilke Adalioglu,2023[17]
**KNN**	93.13%	80.83%	87.50%	89.93%	88.53%	Fused A2C & A4C	Our proposed method
**DT**	83.03%	74.03%	83.03%	81.71%	79.59%		
**RF**	87.14%	77.33%	87.14%	86.19%	84.25%		
**SVM**	93.13%	83.61%	88.52%	90.39%	89.25%		
**MLP**	80.60%	81.40%	79.83%	80.24%	82.21%		

The SVM classifier in our study also outperforms in terms of mean sensitivity (95.00%) and specificity (79.07%) compared to work reported by Aysen Degerli‬, 2021 with a sensitivity of 83.09% and specificity of 74.03%[5]. Our approach also showcases competitive results against methods like Echo cardio 3D-Net (EC3D-Net) (AUC = 82%) [34], R3D-18 (ACC = 80.22%) [19], and LVSnake (GCN-based) [16], indicating the robustness of our classifier. In comparison with all 6 other studies using the HMC-QU dataset, our model showed the best performance in predicting the outcome with the best accuracy 89.25%, while the best of others is 85.00%.

Our study evaluated the performance of the optical flow motion field features on SVM and KNN classifiers using the HMC-QU dataset, rigorously applying a stratified 5-fold CV process to ensure reliability. A distinctive feature of our methodology is the utilization of multi-view echocardiograms, specifically from the A4C and A2C views, which significantly enhance diagnostic accuracy compared to the single-view A4C analysis commonly employed in previous research. The A4C and A2C echocardiograms are integral tools in the hands of cardiologists for assessing LV wall motion. One pivotal methodological decision that significantly improved our results was the concatenation of extracted features from both the A2C and A4C views for RWMA classification. In Tables 5 and 6, individual view performance results of these echocardiograms revealed lower performance in comparison to when multiple views were aggregated. When analyzed individually, the results from A2C and A4C views were found to be subpar. However, combining the feature sets from both views proved to improve the classifiers’ performance. The aggregated view or multi-view approach additional information present in the A2C and A4C views. This aligns with the cardiologists’ practice, who often take into account multi view distinct motion attributes in the A4C and A2C echocardiograms, bridging the gap between computational analysis and clinical observation.

In summary, by integrating sophisticated ML techniques and leveraging optical flow motion field features from the segmented multi-view (A4C and A2C) echocardiograms, our study effectively addresses the limitations commonly observed in prior research that used only single-view (A4C) echocardiograms. We offer significant advancements in both the scientific rigor and clinical applicability of RMWA diagnostics, setting a new standard in the field.

**Fig 8:**
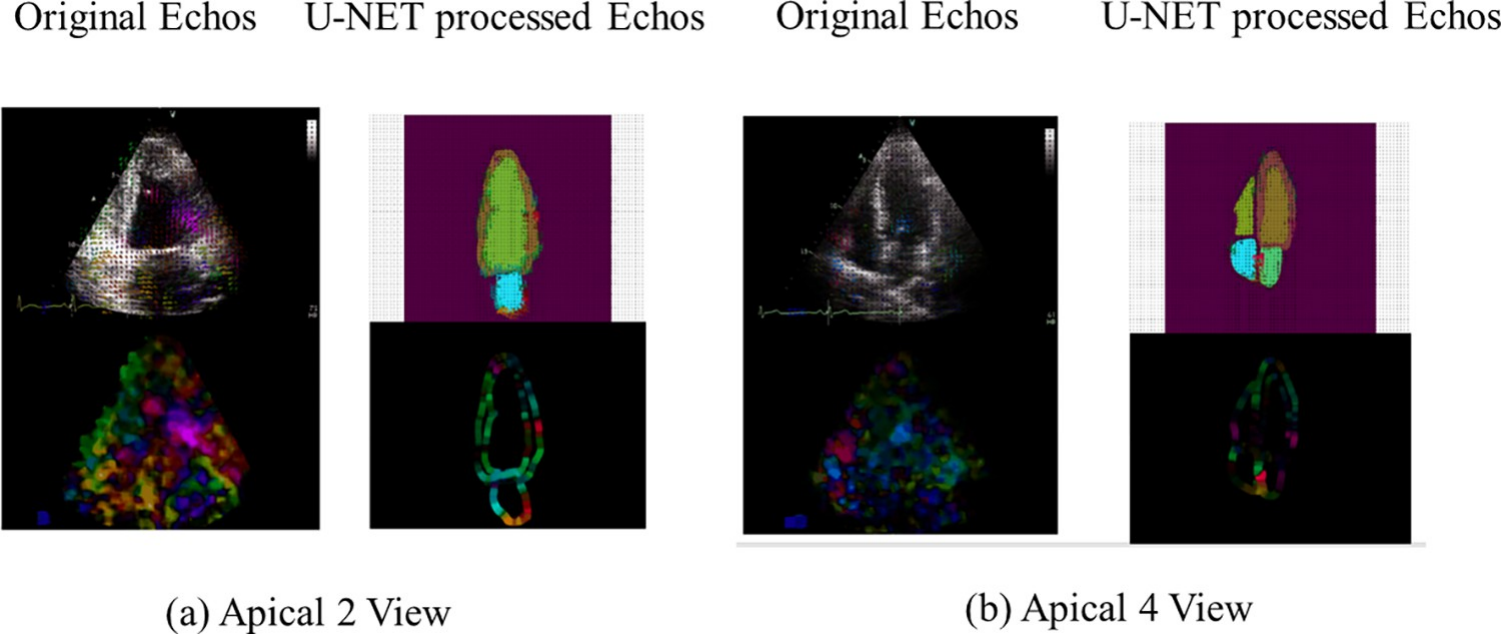
A comparative generated dense optical-flow frames of the original echocardiogram versus the U-Net processed echocardiogram. Representation includes the sample frame of input video, the generated optical flow frame, which are visually enhanced with colour arrows to indicate the direction and magnitude of motion, for both (a) Apical 2 View and (b) Apical 4 View.

Furthermore, the multi-cycle analysis of heart motion, avoids complex preprocessing cardiac cycle frame collection which involves obtaining specific end-systole and end-diastole frames, and marks a significant departure from the typical single-cycle studies [3,5,10,16], allows for a more comprehensive capture of cardiac function over time. The higher performance obtained in this study is also due to improve image quality by removal of noise based on pretrained U-Net in the example echos in Fig 8, and provide better performance than the end-to-end R3D-18 or R(2+1D)-18 algorithms [19,34]. The application of optical flow algorithm had enhanced the motion features by to track cardiac motion from echocardiogram tissue velocity images [11]. Our study’s use of the temporal ConvNet module further extends the capability to examine a broader spectrum of motion features. This extension goes beyond the conventional reliance on time series image features or the peak values of LV contour segment displacement.

This study acknowledges several limitations, primarily concerning the HMC-QU dataset. The dataset is restricted to two significant yet specific echocardiographic views. This limitation potentially compromises the comprehensiveness and generalizability of our findings. To mitigate this issue, future research will aim to integrate more diverse datasets that include a broader spectrum of echocardiographic views. This expansion would provide a more comprehensive understanding, potentially unveiling new insights and enhancing the robustness of our results. Our objective is to elevate the efficiency and accuracy of our system, particularly in real-time scenarios. This approach will probably improve with further training on new datasets and the addition of echocardiography views (eg, the Apical 3 Chamber (A3C), Apical 5 Chamber (A5C), Parasternal Long Axis (PLAX), and Parasternal Short Axis (PSAX) views.)). With development and refinement, this method could be used clinically to provide superior, more individualized, survival predictions.

## Conclusion

Our research introduces a novel approach to detecting Regional Wall Motion Abnormalities (RWMA) using echocardiograms, featuring several innovative aspects compared to existing studies. The multi-cycle analysis of heart motion, diverging from the more common single-cycle echocardiographic studies, allows for a more comprehensive capture of cardiac function over time. The integration of U-Net for precise cardiac structure segmentation, coupled with optical flow algorithms and Temporal Convolutional Networks, presents a methodologically advanced technique for detailed cardiac wall motion feature extraction. Additionally, the employment of both machine learning and deep learning classifiers provides a robust mechanism for accurate RWMA prediction.
